# Editorial: The Role of Systemic and Cellular Metabolism on Susceptibility to Infections and Responsiveness to Vaccination

**DOI:** 10.3389/fcimb.2022.854241

**Published:** 2022-03-04

**Authors:** Antonella Caputo, Carlos A. Guzman, Clovis S. Palmer, Francesco Nicoli

**Affiliations:** ^1^ Department of Chemical, Pharmaceutical and Agricultural Sciences, University of Ferrara, Ferrara, Italy; ^2^ Department Vaccinology and Applied Microbiology, Helmholtz Centre for Infection Research, Braunschweig, Germany; ^3^ Division of Comparative Pathology, Tulane National Primate Research Center, Covington, LA, United States

**Keywords:** cellular metabolism, immunometabolism, non-communicable diseases, metabolic disorder, bacteria, virus, pathogen

The severity of bacterial and viral infections is linked to impaired immune and metabolic disorders, disproportionally affecting individuals with metabolic comorbidities such as obesity, type 2 diabetes, and cardiovascular diseases. These metabolic disorders are also key predictors of adverse outcomes to infections. Infections by themselves promote both cellular and systemic metabolic alterations resulting in the development of long-term comorbidities. Indeed, the recent SARS-CoV-2 pandemic highlighted this important connection, since metabolic complications emerged as key risk factors for the development of severe forms of COVID-19 disease (Steenblock et al., 2021). However, the precise molecular determinants of these associations remain unclear. A greater understanding on how biochemical processes connect disease outcomes in viral and bacterial infections will facilitate the development of more effective host-directed interventions and diagnostic tools for patient stratification or assessment of disease progression. Therefore, this Research Topic aimed at highlighting work that: (1) disentangles how intrinsic immune cellar metabolic processes control viral replication and immunity, (2) dissect the metabolic requirements of immune cells during bacterial infections, and (3) examine systemic metabolic dysregulation during viral infections.

## Host Cell Metabolism and Pathogen Replication

Viruses hijack host metabolic machineries for the synthesis of macromolecules, such as lipids, proteins, and nucleic acids essential for viral replication and productive infection. The tricarboxylic acid (TCA) cycle and mitochondria have established roles in biogenesis and adenosine triphosphate (ATP) production. Beyond this, Sánchez-García et al. persuasively discussed critical immunoregulatory roles of the TCA cycle. Viruses may take advantage of TCA intermediates such as citrate for their replication processes. As such, citrate is not only critical for *de novo* lipid synthesis for viral replication but partakes in important epigenetic reprogramming (e.g., acetylation) which controls viral latency. Direct inflammatory roles of citrate and succinate have also been demonstrated. Conversely, viral infections may promote synthesis of anti-inflammatory and anti-viral metabolites such as itaconate and fumarate. In particular, fumarate and its derivative dimethyl fumarate are currently being exploited therapeutically for their anti-inflammatory properties – particularly to tame inflammation driven by M1-like glycolytic macrophages.

Further studies are therefore warranted to assess whether pathogens, in addition to exploiting host metabolites as building blocks for nascent virions, may also subvert cell metabolic processes to their advantage as an immune evasion strategy (e.g., by shifting from citrate catabolism toward itaconate-mediated anabolic reactions).

## Host Cell Intrinsic Metabolism During Infection – Therapeutic Opportunities

The essential role of the TCA and its metabolites in viral infections reviewed by Sánchez-García et al. illustrates the current shift in our understanding of the importance of host metabolic factors in inflammatory responses and viral control. This has been exploited to identify new classes of therapeutics to limit inflammatory and metabolic-associated diseases linked to various infections. A similar approach was recently proposed for SARS-CoV-2 (Olagnier et al., 2020). Glucose and amino acid metabolism intricately link pathogen replication and inflammatory responses. However, pathogens may interact with several other host biochemical pathways, and each of them may represent a potential target for a therapeutic strategy. Sequestration of iron from bacterial pathogens is an important host mechanism to control infection. As such, in this Research Topic, Hoffmann et al. exploited iron metabolism as a therapeutic target for bacterial infections. They inhibited hepcidin, which degrades the cellular iron exporter ferroportin, that is upregulated during bacterial infections. Hepcidins increases iron retention in macrophages, thereby limiting circulating iron for pathogens. However, this mechanism could also increase iron availability for intracellular bacteria, becoming beneficial for the pathogen and detrimental for the host. To interrogate these scenarios Hoffmann et al. used two hepcidin inhibitors that showed encouraging effects *in vitro*, but were neither effective in modulating hepcidin and iron levels in infected animals nor influenced bacterial burden. Whether the biological properties and *in vivo* activity of the inhibitors can be improved by using medicinal chemistry remains to be elucidated. Nevertheless, despite their therapeutic failure, the approach highlights the challenges to overcome metabolic redundancy. Indeed, they argue that host cells and microbes could engage several compensatory mechanisms once a biochemical pathway is inhibited. This view as well as the possibility of triggering – depending on the pathogen – opposing effects warrants consideration when targeting metabolic pathways as a therapeutic strategy.

## Systemic Metabolic Homeostasis in Infections – Opportunities for Biomarker Discovery

Pathogens may not only exploit intrinsic cellular metabolism in host cells but impact systemic metabolic homeostasis that underpins the development of comorbidities. Dysregulated whole body lipid homeostasis in HIV+ adults - irrespective of anti-retroviral treatment (ART) - is a hallmark of chronic HIV infection, and is associated with the development of metabolic comorbidities (Godfrey et al., 2019). However, it is less clear how this scenario plays out in HIV+ children. Studying a cohort of 5-10-year-old Tanzanian children, Mbuya et al. found significant lipid alterations among ART-treated HIV+ children. While the lack of cardiovascular disease measurements precludes the examination of a relationship with childhood dyslipidemia in this cohort, such a lipid profile has been associated with atherosclerosis risks in HIV+ adults. Although Mbuya et al. showed a reduced vaccine responsiveness in HIV+ children, they found no correlation between changes in lipid profile, proinflammatory cytokine levels, and humoral responses to vaccines. Nonetheless, the conceptualization by Mbuya et al. that suggests that dyslipidemia driven by HIV and/or ART itself could impact vaccine-induced immunity against other infection warrants future studies.

Serum levels of metabolites - including those of the TCA cycle - have been proposed for the diagnosis of infections, as well as prognostic factors for the development of severe COVID-19 and long COVID (Giron et al., 2021; Shi et al., 2021). Another encouraging prognostic biomarker for COVID-19 has been identified by Chauvin et al., showing that serum neopterin levels at hospital admission are predictive of extended symptomatic disease and poor-survival post SARS-CoV-2 infection. Neopterin belongs to the family of pteridines. It is derived *in vivo* from guanosine triphosphate (GTP) through a reaction occurring in activated immune and endothelial cells upon stimulation. Therefore, its levels likely reflect an inflammatory status. The analysis of 374 COVID-19 patients showed that those who died during the study period presented with elevated levels of neopterin. This was among the strongest discriminant parameters between COVID-19 patients who recovered or died also when compared to other risk factors such as increased age, hypertension, diabetes, and cardiovascular disease. The authors set a cut-off value of 53 nM and showed that SARS-CoV-2 patients with systemic neopterin levels higher than the cut-off had a remarkably 13-fold higher risk of death. Chauvin et al. also showed that systemic neopterin levels are strong predictors of SARS-CoV-2 infection per se, regardless of fatality outcomes. Arguably, neopterin levels may increase our understanding of the metabolic comorbidities that increase predisposition to SARS-CoV-2 infection. Notwithstanding, other works confirmed the predictive value of neopterin in severe SARS-CoV-2 infection (4). Further studies aimed at assessing its potential use as a universal prognostic biomarker in other hyperinflammatory conditions are needed.

## Conclusions

The four papers presented in this Research Topic show that viral and bacterial infections exploit the full biochemical arsenal of the host and may affect the levels of various metabolites. The biochemical interactions between pathogens and their host is a very complex phenomenon made of redundant pathways and reciprocal modulations, which may be influenced by external factors, such as diet and lifestyle. This will certainly render the translational transfer of potential targets from mechanistic studies to effective therapeutic approaches difficult. Nonetheless, an iterative refinement process can speed up discovery and transfer into the clinical pipeline. Moreover, these studies have shown the strong potential for the identification of biomarkers to develop point-of-care diagnostics for disease outcomes.

## Author Contributions

All authors listed have made a substantial, direct, and intellectual contribution to the work, and approved it for publication.

## Conflict of Interest

The authors declare that the research was conducted in the absence of any commercial or financial relationships that could be construed as a potential conflict of interest.

## Publisher’s Note

All claims expressed in this article are solely those of the authors and do not necessarily represent those of their affiliated organizations, or those of the publisher, the editors and the reviewers. Any product that may be evaluated in this article, or claim that may be made by its manufacturer, is not guaranteed or endorsed by the publisher.

## References

[B1] Giron L. B. Dweep H. Yin X. Wang H. Damra M. Goldman A. R.. (2021). Plasma Markers of Disrupted Gut Permeability in Severe COVID-19 Patients. Front. Immunol. 12, 686240. doi: 10.3389/fimmu.2021.686240 34177935 PMC8219958

[B2] Godfrey C. Bremer A. Alba D. Apovian C. Koethe J. R. Koliwad S.. (2019). Obesity and Fat Metabolism in Human Immunodeficiency Virus-Infected Individuals: Immunopathogenic Mechanisms and Clinical Implications. J. Infect. Dis. 220, 420–431. doi: 10.1093/infdis/jiz118 30893434 PMC6941618

[B3] Olagnier D. Farahani E. Thyrsted J. Blay-Cadanet J. Herengt A. Idorn M.. (2020). SARS-CoV2-Mediated Suppression of NRF2-Signaling Reveals Potent Antiviral and Anti-Inflammatory Activity of 4-Octyl-Itaconate and Dimethyl Fumarate. Nat. Commun. 11, 4938. doi: 10.1038/s41467-020-18764-3 33009401 PMC7532469

[B4] Shi D. Yan R. Lv L. Jiang H. Lu Y. Sheng J.. (2021). The Serum Metabolome of COVID-19 Patients Is Distinctive and Predictive. Metabolism 118, 154739. doi: 10.1016/j.metabol.2021.154739 33662365 PMC7920809

[B5] Steenblock C. Schwarz P. E. H. Ludwig B. Linkermann A. Zimmet P. Kulebyakin K.. (2021). COVID-19 and Metabolic Disease: Mechanisms and Clinical Management. Lancet Diabetes Endocrinol. 9, 786–798. doi: 10.1016/S2213-8587(21)00244-8 34619105 PMC8489878

